# The *Nrf2*-Antioxidant Response Element Signaling Pathway Controls Fibrosis and Autoimmunity in Scleroderma

**DOI:** 10.3389/fimmu.2018.01896

**Published:** 2018-08-16

**Authors:** Niloufar Kavian, Souad Mehlal, Mohamed Jeljeli, Nathaniel Edward Bennett Saidu, Carole Nicco, Olivier Cerles, Sandrine Chouzenoux, Anne Cauvet, Claire Camus, Mehdi Ait-Djoudi, Christiane Chéreau, Saadia Kerdine-Römer, Yannick Allanore, Frederic Batteux

**Affiliations:** ^1^Laboratoire d’Immunologie, Hôpital Cochin, Paris, France; ^2^INSERM U1016, Institut Cochin, Paris, France; ^3^UMR996 – Inflammation, Chemokines and Immunopathology, INSERM, Univ Paris-Sud, Université Paris-Saclay, Châtenay-Malabry, France; ^4^Service de Rhumatologie, Hôpital Cochin, Paris, France

**Keywords:** systemic sclerosis, oxidative stress, fibrosis, inflammation, Nrf2

## Abstract

Systemic sclerosis (SSc) is an autoimmune disease with fibrosis of the skin and internal organs and vascular alterations. Dysregulations in the oxidant/antioxidant balance are known to be a major factor in the pathogenesis of the disease. Indeed, reactive oxygen species (ROS) trigger neoepitopes leading to a breach of immune tolerance and autoimmune responses, activate fibroblasts to proliferate and to produce excess of type I collagen. ROS also alter endothelial cells leading to vascular dysfunction. Glutathione (GSH) is the most potent antioxidant system in eukaryotic cells. Numerous studies have reported a defect in GSH in SSc animal models and humans, but the origin of this defect remains unknown. The transcription factor NRF2 is a key player in the antioxidant defense, as it can induce the transcription of antioxidant and cytoprotective genes, including GSH, through its interaction with the antioxidant response elements. In this work, we investigated whether NRF2 could be implicated in the pathogenesis of SSc, and if this pathway could represent a new therapeutic target in this orphan disease with no curative medicine. Skin biopsies from 11 patients and 10 controls were harvested, and skin fibroblasts were extracted. Experimental SSc was induced both in BALB/c and in *nrf2*^−/−^ mice by daily intradermal injections of hypochloric acid. In addition, diseased BALB/c mice were treated with an *nrf2* agonist, dimethyl fumarate, or placebo. A drop in *nrf2* and target genes mRNA levels was observed in skin fibroblasts of SSc patients compared to controls. Moreover, the *nrf2* pathway is also downregulated in skins and lungs of SSc mice. In addition, we observed that *nrf2*^−/−^ mice have a more severe form of SSc with increased fibrosis and inflammation compared to wild-type SSc mice. Diseased mice treated with the *nrf2* agonist dimethyl fumarate (DMF) exhibited reduced fibrosis and immune activation compared to untreated mice. The *ex vivo* treatment of skin fibroblasts from SSc mice with DMF restores GSH intracellular content, decreases ROS production and cell proliferation. These results suggest that the *nrf2* pathway is highly dysregulated in human and SSc mice with deleterious consequences on fibrosis and inflammation and that Nrf2 modulation represents a therapeutic target in SSc.

## Introduction

Systemic sclerosis (SSc) is a rare and severe connective tissue disorder characterized by progressive fibrosis of the skin and visceral organs due to excessive collagen deposition, vasculopathy, and autoimmunity. Skin sclerosis and Raynaud’s phenomenon are the main clinical features that exhibit a strong impact on quality of life, whereas development of pulmonary fibrosis is life-threatening and intractable (1, 2). Even though the pathogenesis of the disease is unclear, it is well admitted that abnormalities in fibroblasts, endothelial cells, and immune cells lead to the fibrotic, vascular, and autoimmune processes (3). In addition, the role of oxidative stress in SSc has also been highlighted by many studies in animal models and in patients (4–6). Noticeably, skin fibroblasts from SSc patients and mice spontaneously produce high amounts of reactive oxygen species (ROS) that contribute to fibroblasts activation and proliferation as well as collagen synthesis (6, 7). In earlier works, we have developed an animal model of SSc induced by intradermal injections of HOCl, a substance generating ROS *in vivo*, demonstrating the direct role of ROS in the breach of immune tolerance, thus participating to the pathogenesis of the disease (4). Subsequently, the benefit of reducing ROS production by fibroblasts, immune cells, and endothelial cells for the clinical improvement of the disease has been underlined (8–10). Antioxidant defenses now appear to be crucial in SSc development in regulating excessive ROS production, and play a key role in the pathogenesis of the disease (11–13). Among them, the nuclear factor erythroid 2 (NF-E2)-related factor 2 (Nrf2) is a key cellular sensor of oxidative stress that can induce the transcription of cytoprotective genes protecting cells from excessive oxidative stress. At physiological levels and in the absence of major cellular stresses, NRF2 is linked to Keap1 in the cytoplasm. When ROS overcomes the endogenous antioxidant capacity, Keap 1 releases NRF2 which then translocates into the nucleus where it binds the antioxidant response elements in association with other transcription factors and accessory proteins. This event causes the transcriptional activation of major key antioxidants and cytoprotective proteins and enzymes responsible, among others, of glutathione (GSH) synthesis. Recent studies have reported a dysregulation in the Nrf2 pathway in a wide area of pathologies including cancers, inflammatory, and fibrotic diseases (14–17). Interestingly, dimethyl fumarate (DMF), a molecule, which at certain concentrations enhances Nrf2 activity, has shown remarkable beneficial effects in two autoimmune diseases: multiple sclerosis and psoriasis (18, 19). Based on the roles that ROS play in SSc, we hypothesized that Nrf2 could be involved in the pathogenesis of SSc and therefore be an interesting target for the treatment of this orphan disease. In the present work, we show that the *nrf2* pathway is dysregulated in patients and in mice with SSc, and report a more severe form of SSc in Nrf2^−/−^ mice along with the therapeutic properties of the *nrf2* agonist DMF.

## Materials and Methods

### Patients

Total mRNA issued from resting cultured fibroblasts derived from 11 (*n* = 11) SSc patients and 10 (*n* = 10) healthy subjects were kindly provided by Pr. Yannick Allanore, Rheumatology Department, Cochin Hospital, Paris, France. All the patients gave their informed consent and their clinical features are represented in **Table 1**.

**Table 1:** Characteristics of systemic sclerosis (SSc) patients and healthy subjects.

Characteristics	Diffuse SSc	Controls
Number of subjects	*n* = 11	*n* = 10
Sex (female/male)	10/1	8/2
Age mean (min–max)	56.3 (26–72)	30 (15–45)
Biospy site	Forearm fibrotic skin	Forearm normal skin
Duration of disease in months mean (min–max)	51 (3–210)	–
Rodnan score mean (min–max)	17.5 (5–29)	–
Autoantibodies	7 anti-topoisomerase I (Scl70) 3 ANA with no specificities	–
Lung fibrosis	6	–
Pulmonary arterial hypertension	2	–
**Treatments**		
Steroids	6	–
Methotrexate	4	–
Rituximab	2	–
Tocilizumab	1	–
Azathioprine	1	–

Sex, age at time of biopsy, SSc type, disease duration, biopsy site, treatments, Rodnan score, presence/absence of pulmonary arterial hypertension, and autoantibodies were collected for each patient included in this study and reported in this table.

### Mice

Six-week-old female BALB/c mice purchased from Janvier Laboratory (Le Genest Saint Isle, France) were used for the induction of experimental SSc. Nrf2^−/−^ mice and control wild-type mice were a generous gift from S. Kerdine-Römer and were previously described (20). Control mice for all experiments were age-, sex-, and weight-matched. All animals were given human care according to the guidelines of our institution. The project was approved by the approval of the Regional Ethic Committee on Animal Experimentation under the number CEEA34.CN.023.11.

### *In Vivo* Induction of Experimental SSc and Treatments

Two experimental SSc mice models were used. HOCl-induced SSc (HOCl-mice) developed following daily intradermal injections of 200 µl of HOCl-generating reagents into the back of BALB/c mice for 6 weeks, as previously described (21). Bleomycin-induced SSc developed following daily intradermal injections of 100 µl bleomycin (100 µg/ml) in phosphate buffered saline (PBS), for 6 weeks (bleomycin-mice) (22). Control groups received injections of 200 µl sterilized PBS. After 6 weeks, all the animals were sacrificed by cervical dislocation. Lungs were collected and skin biopsies were performed on the back region with a punch (6 mm diameter). Samples were stored at −80°C for western-blot, mRNA quantification, determination of collagen content, or fixed in 10% formalin for histopathological analysis. Experimental *in vivo* mice experiments were performed twice.

### DMF Treatment

Mice were given oral 25 mg/kg/day of DMF (Sigma Aldrich, St. Quentin Fallavier, France) by gavage every day for 6 weeks.

### Cell Lines and Primary Fibroblasts From Mouse and Humans

Human pulmonary microvascular endothelial cells and human venal endothelial cells (HUVECs) were purchased from Promocell (Heidelberg, Germany) and NIH-3T3 (mouse fibroblasts) were obtained from the American Type Culture Collection (Manassas, VA, USA). Murine control and SSc primary skin fibroblasts were isolated from mouse skin as previously described (23). Human primary skin fibroblasts were isolated from punch biopsies from SSc patients and control subjects. Briefly, 4 mm punch biopsies were collected from patients (forearm fibrotic skin) and immediately diced with scalpels in collagenase for 2 h at 37°C. Cells were then rinsed, filtered, and cultured in complete DMEM in T25 at 37°C. Fibroblasts were observed after 3–5 days and expanded.

### Measurement of Intracellular Levels of GSH, and of H_2_O_2_ Released by Endothelial Cells and Fibroblasts *In Vitro*

Fibroblasts and endothelial cells are seeded in triplicates in 96-well microplates (2 × 10^4^ cells/well) and incubated with complete medium for 24 h at 37°C. Increasing amounts of DMF (0–25 µM) were added to the cells. Levels of H_2_O_2_ and GSH were assessed by spectrofluorometry (Fusion, PerkinElmer, Wellesley, MA, USA) using 200 µM 2′,7′-dichlorodihydrofluorescein diacetate (H2DCFDA) or 50 µM monochlorobimane (both from Molecular Probes, The Netherlands), respectively, in PBS for 30 min at 37°C. Fluorescence intensity was read every hour for 6 h. The numbers of viable cells were evaluated by crystal violet assay as previously described (23). Results were expressed as arbitrary units of fluorescence intensity per million of viable cells.

### Reagents

Dimethyl fumarate and all other reagents were purchased from Sigma Aldrich (St. Quentin Fallavier, France).

### Assays for Anti-DNA Topoisomerase 1 Autoantibodies and Proinflammatory Cytokines in the Sera

Levels of anti-DNA topoisomerase 1 IgG antibodies (Abs) were detected by enzyme-linked immunosorbent assay (ELISA) using purified calf thymus DNA topoisomerase I bound to the wells of a microtiter plate (Abnova, Germany). A 1:4 mice serum dilution and a 1:1,000 anti-murine Ig HRP (DAKO) secondary antibody dilution were used.

INFγ and IL-13 in the sera were also measured by ELISA using Mouse ELISA Ready-SET-Go (eBioscience-Thermo Scientific), following the manufacturer’s instructions.

### Assessment of Skin Thickness and Collagen Accumulation in Skin and Lungs

Skin thickness of the shaved back of mice was measured 1 day before sacrificing the mice with a caliper and expressed in millimeters. Fixed lung and skin pieces were embedded in paraffin. A 5-μm-thick tissue section was prepared from the mid-portion of paraffin-embedded tissue and stained with H&E. Slides were examined by standard brightfield microscopy (Nikon Eclipse 80i) (Nikon, Champigny sur Marne, France) by a pathologist who was blinded to the assignment of the animal to an experimental or a control group.

Determination of collagen content in skin samples was performed by the hydroxyproline assay. After digestion of punch biopsies (3 mm) in 6 M HCl for 3 h at 120°C, the pH of the samples was adjusted to 7 with 6 M NaOH. Samples were then mixed with 0.06 M chloramine T and incubated for 20 min at room temperature. Next, 3.15 M perchloric acid and 20% p-dimethylaminobenzaldehyde were added and samples were incubated for an additional 20 min at 60°C. The absorbance was determined at 557 nm.

### FACS Analysis of T Lymphocytes and Macrophages Subpopulations

Cell suspensions from mouse spleens were prepared after hypotonic lysis of erythrocytes in potassium acetate solution. Cells were incubated with the appropriate labeled antibody cocktail (ebioscience, ThermoFisher Scientific) at 4°C for 30 min in PBS with 0.1% sodium azide and 5% normal rat serum. Flow cytometry was performed on a FACSCanto flow cytometer (BD Biosciences) using standard techniques. The monoclonal Abs used in this study were anti-CD3-FITC, anti-CD4-BV421, anti-CD8-PE-Cy7, anti-CD69-PE, and anti-B220-APC in a first mix. In a second mix, the mAbs used were anti-B220-BV421, anti-CD11b-PerCP-Cy5.5, anti-Ly6C-PE-Cy7, and anti-CD62L-APC. M1 macrophages were defined as B220^−^CD11b^+^Ly6c^+^CD62L^+^ and M2 macrophages as B220^−^CD11b^+^Ly6c^−^CD62L^−^. Data were analyzed with the FlowJo software (Tree Star).

### Western-Blot Analysis in Mouse Fibroblasts

Fibroblasts isolated as forementioned were incubated with 50 µl RIPA. Protein extracts (30 µg total proteins) were subjected to 10% polyacrylamide gel electrophoresis, transferred onto nitrocellulose membranes, blocked with 5% non-fat dry milk in Tris Buffer Solution-Tween, then incubated overnight at 4°C with an anti-NRF2 antibody (1:500, Santa Cruz, sc-722). The membranes were washed and incubated with an HRP-conjugated secondary antibody (Santa Cruz, Paris, France) for 1 h at room temperature. Immunoreactivities were revealed with ECL (Amersham).

### RT-qPCR Analysis

Total murine RNA was extracted from crushed samples using the RNeasy mini kit (Qiagen, France). One-step RT-qPCR was performed using QuantiTect SYBR^®^ Green RT-PCR Kit on a LightCycler 480 II instrument (Roche Applied Science, France). The sequences of the primers are detailed in Table S1 in Supplementary Material. Samples were normalized to mRNA expression of housekeeping genes (HRPT2 for murine RNA and GAPDH for human ones), and results were provided either as relative expression to these housekeeping genes using the formula 2^−ΔCt^ and as fold increase using the formula 2^−ΔΔCt^. RT-PCR was carried out for 40 cycles, with a denaturing phase of 15 s at 94°C, an annealing phase of 30 s at 60°C, and an extension phase of 30 s at 72°C.

### Statistical Analysis

Microsoft Excel 2007 and GraphPad Prism (GraphPad Inc., USA) softwares were used to analyze the data. All values are averages of at least two independent experiments made in triplicates, except when specified. Error bars shown in the figures represent SEM and all results were expressed as arithmetic mean ± SEM. Differences between the experimental groups were analyzed using Mann–Whitney *U* test, statistically significant differences were reported as follows: **p < 0.01 or *p < 0.05.

## Results

### The Nrf2 Pathway Is Downregulated in SSc Patients’ Fibroblasts

**Figure 1:**
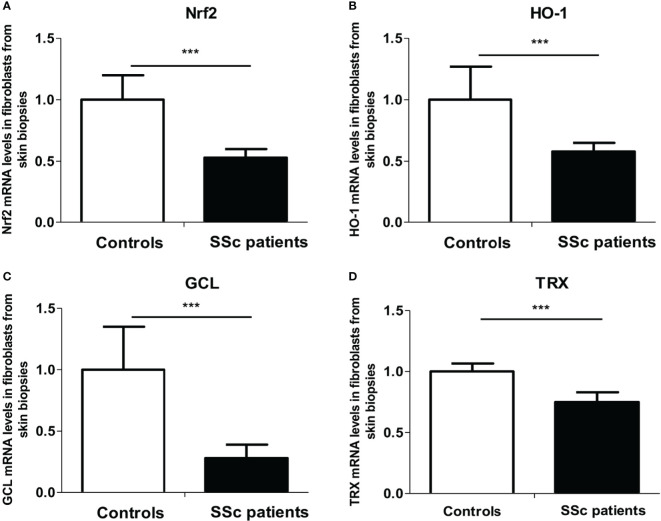
The *nrf2* pathway is downregulated in the skin of systemic sclerosis (SSc) patients. **(A)** *Nrf2* mRNA levels in the fibroblasts from skin of control subjects and SSc patients. **(B)** Heme oxygenase-1 (HO-1) mRNA levels in the fibroblasts from skin of control subjects and SSc patients. **(C)** Glutamate-cystein ligase (GCL) mRNA levels in fibroblasts from skin of control subjects and SSc patients. **(D)** Thioredoxin (TRX) mRNA levels in the fibroblasts from the skin of control subjects and SSc patients. Data from 13 SSc patients and 10 control subjects. Values are mean ± SEM. ***p ≤ 0.001, by Mann–Whitney *U* test.

As ROS metabolism is impaired in the skin of SSc patients, we first investigated the Nrf2 pathway in fibroblasts extracted from the skin of patients. We analyzed the mRNA expression levels of *nrf2* and its main target genes involved in the regulation of ROS production [heme oxygenase-1 (HO-1), glutamate cysteine ligase (GCL), and thioredoxin (TRX)] by quantitative RT-PCR in fibroblasts extracted from skin biopsies from SSc patients and healthy controls. We showed a highly significant downregulation of *nrf2* (*p* = 0.0006, **Figure 1A**) and *nrf2*-induced genes mRNA levels in patients’ skin fibroblasts, HO-1 (*p* = 0.001, **Figure 1B**), GCL (*p* < 0.0001, **Figure 1C**), and TRX (*p* < 0.0001, **Figure 1D**) compared to healthy individuals.

### The Nrf2 Pathway Is Downregulated in the Skin of HOCl-Mice

**Figure 2:**
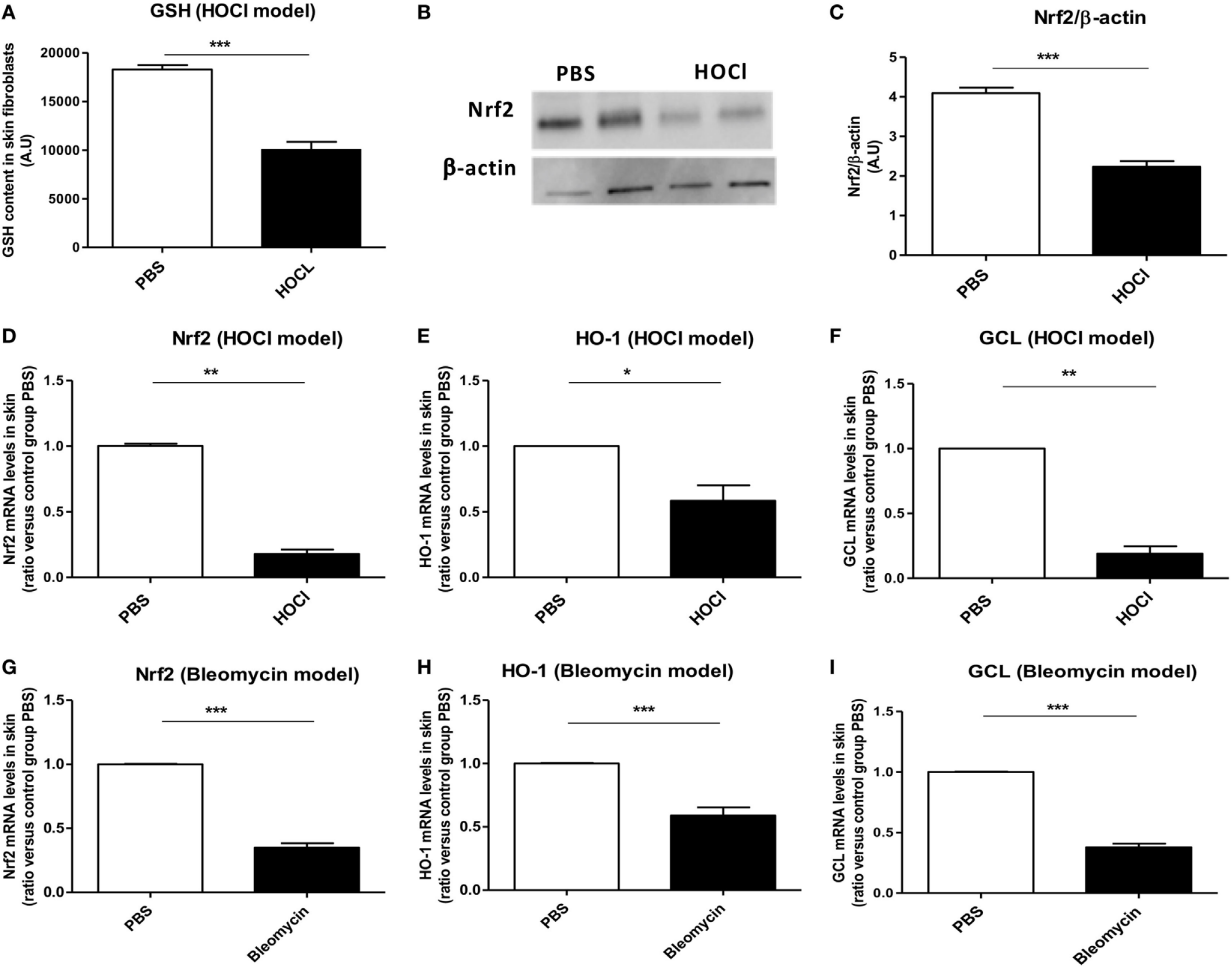
The *Nrf2* pathway is downregulated in the skin of mice with SSc. **(A)** GSH content in skin fibroblasts from PBS (controls) and HOCl-mice (SSc mice) (A.U per cells). **(B)** Protein expression levels of NRF2 in PBS and HOCl in skin extracts by western-blot (two mice representative of seven). Photographs were taken with a Fujifilm LAS-3000. **(C)** Intensity ratio of Nrf2 and β-actin expression in skin in PBS- and HOCl-mice. **(D–F)** The Nrf2 pathway is downregulated in the skin of mice with ROS-induced SSc. mRNA levels of *Nrf2* **(D)**, HO-1 **(E)**, and GCL **(F)** mRNA measured in skin extracts. **(G–I)** The Nrf2 pathway is downregulated in the skin of mice with bleomycin-induced SSc. Levels of NRF2 **(G)**, HO-1 **(H)**, and GCL **(I)** mRNA measured in skin extracts. Abbreviations: AU, arbitrary units; SSc, systemic sclerosis; GSH, glutathione; PBS, phosphate buffered saline; ROS, reactive oxygen species; HO-1, heme oxygenase-1. Values are mean ± SEM (*n* = 8 mice per group). *p ≤ 0.05; **p ≤ 0.01; ***p ≤ 0.001, by Mann–Whitney *U* test.

Glutathione is an essential cofactor of ROS catabolism and its dysregulation can lead to uncontrolled production of ROS, as it is observed in the mouse model of HOCl-induced SSc (8, 24). As previously described (8), fibroblasts from HOCl-mice display a severe reduction in the levels of reduced GSH of more than 40% compared to those from control PBS mice (*p* = 0.003, **Figure 2A**).

Since Nrf2 controls the transcription of GCL, a key enzyme in the synthesis of GSH, we therefore, in this study examined the protein expression levels of NRF2 in the skin of HOCl-mice. We observed a reduction in the NRF2 protein levels in HOCl-fibroblasts compared to PBS-fibroblasts (*p* = 0.0002 **Figures 2B,C**). This result correlated with the mRNA levels of *nrf2* that were also strongly diminished in diseased skin fibroblasts from HOCl-mice compared to those from PBS mice (*p* = 0.008, **Figure 2D**). Furthermore, the mRNA expression levels of GCL and HO-1 were downregulated in diseased HOCl-fibroblasts compared to control PBS-fibroblasts (*p* = 0.02 and *p* = 0.004, respectively, **Figures 2E,F**). No difference in TRX mRNA expression levels between normal and disease fibroblasts was, however, observed; but expression levels of TXNIP, a natural inhibitor of TRX, were dramatically increased in HOCl-SSc mice compared to controls (data not shown).

The same phenomenon was observed in bleomycin-treated SSc mice with a very significant downregulation in *nrf2* and related genes mRNA levels compared to PBS mice (*p* = 0.0004, *p* = 0.0002, *p* < 0.0001, respectively, **Figures 2G–I**).

### Nrf2^−/−^ HOCl-Mice Display Severe Dysregulations in the Redox Balance

**Figure 3:**
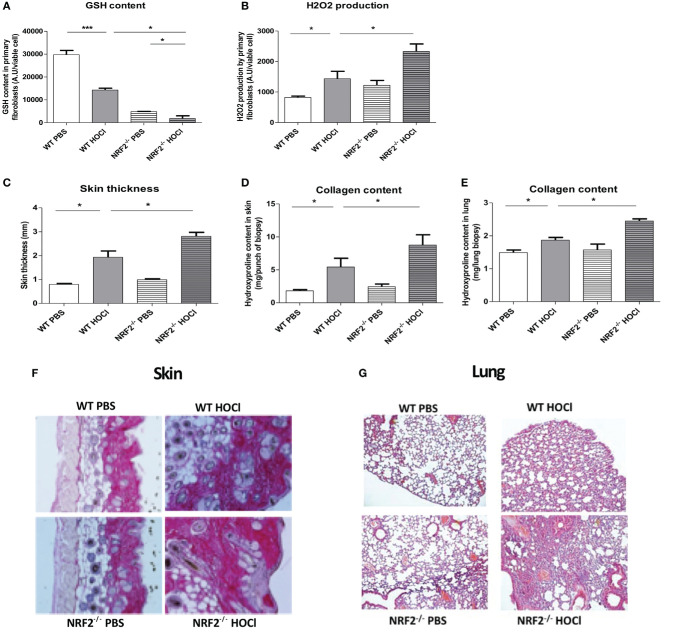
*nrf2*^−/−^ mice exposed daily to HOCl display a severe phenotype of SSc. WT or *nrf2*^−/−^ mice were injected daily with HOCl for 6 weeks. **(A,B)** Measurements of oxidative stress markers in the skin of PBS and HOCl-mice with WT and *nrf2*^−/−^ background. **(A)** GSH content in primary skin fibroblasts (AU per viable cells). **(B)** H_2_O_2_ production by primary skin fibroblasts (AU per viable cells). Fibrosis markers in PBS and HOCl-mice with WT and *nrf2*^−/−^ background. **(C)** Skin thickness in millimeters. **(D)** Collagen content in the skin (Hydroxyproline, mg per punch biospy). **(E)** Collagen content in lung (Hydroxyproline, mg per lung biopsy). **(F)** Skin biopsies stained with picro-sirius red. Representative sections of 5 µm. **(G)** Lung biopsies stained with Hematoxylin and eosin (H&E). Photographs were taken with a Nikon Eclipse 80i microscope. Original magnification ×50. **(H–M)** Immunological parameters measured in PBS and HOCl-mice with WT and *nrf2*^−/−^background. **(H–K)** Anti-DNA topoisomerase 1 antibodies levels in the sera (A.U). **(L)** Number of CD4 T lymphocytes. **(M)** Percentages of activated T cells (CD4^+^CD69^+^ lymphocytes). **(N)** Percentages of M2 macrophages (B220^−^CD11b^+^Ly6c^−^CD62L^−^). **(O)** M2-macrophages (B220^−^CD11b^+^Ly6c^−^CD62L^−^)/M1-macrophages (B220^−^CD11b^+^Ly6c^+^CD62L^+^) ratio. **(P)** IL-13 concentration measured in splenic T-cells supernatants by ELISA (pg/ml) **(I–M)**. Abbreviations: AU, arbitrary units; SSc, systemic sclerosis; PBS, phosphate buffered saline; GSH, glutathione; ELISA, enzyme-linked immunosorbent assay. Values in **(A–K)** are mean ± SEM (*n* = 8 mice per group). *p ≤ 0.05; **p ≤ 0.01; ***p ≤ 0.001, by Mann–Whitney *U* test.

Skin fibroblasts from HOCl-treated sclerodermic animals have a clear decrease in intracellular GSH content compared to PBS-treated control mice (*p* = 0.0002). As expected, skin fibroblasts from *nrf2*^−/−^ mice show a severe drop in GSH content compared to wild-type mice (*p* < 0.0001). Moreover, intracellular GSH levels are significantly reduced in fibroblasts from HOCl-treated *nrf2*^−/−^ mice compared to fibroblasts from HOCl-treated WT mice (*p* = 0.01 **Figure 3A**). These dramatic modulations in the intracellular GSH content have important consequences on the production of the highly ROS H_2_O_2_ by fibroblasts. Indeed, measurements of H_2_O_2_ in skin fibroblasts from HOCl-treated *nrf2*^−/−^ mice showed a significant increase of spontaneous H_2_O_2_ production compared to fibroblasts extracted from HOCl-treated WT mice (*p* = 0.04 **Figure 3B**).

### Invalidation of the *nrf2* Gene Exacerbated the Symptoms of SSc in the Mice

We examined the clinical effects of the absence of *nrf2* in the development of SSc. Nrf2^−/−^ mice were exposed to daily injections of HOCl to induce SSc. They showed a more severe form of SSc compared to wild-type SSc mice as demonstrated by the increase of dermal thickness (*p* = 0.02, **Figure 3C**) and collagen type I content in the skin and lungs (*p* = 0.02, **Figures 3D,E**) in comparison to wild-type SSc mice. Histopathological studies of skin and lung section stained with picro-sirius red showed an excess of collagen accumulation in the skin and in the lungs of diseased *nrf2*^−/−^ mice compared to WT HOCl-mice, as shown in **Figure 3F** (skin) and **Figure 3G** (lung).

Altogether, these results strongly suggested a protective role of NRF2 and related proteins in the development of fibrosis in SSc mice.

### *Nrf2* Invalidation Worsens Inflammation and Autoimmunity in SSc Mice

Systemic sclerosis is a systemic autoimmune disease with chronic activation of adaptive immunity by nuclear auto-antigens such as DNA topoisomerase 1 and production of autoantibodies. Anti-DNA topoisomerase 1 Abs were detected in the sera from both WT-HOCl- and *nrf2*^−/−^ HOCl-mice. A significant increase in these autoantibodies was observed in the sera from *nrf2*^−/−^ HOCl-mice compared to WT-HOCl-mice (*p* = 0.02 **Figure 3H**).

Analysis of spleen cell population showed an accumulation of activated CD4^+^ CD69^+^ T cells in *nrf2*^−/−^ HOCl-mice compared to WT HOCl-mice (*p* = 0.01 **Figures 3I,J**).

In addition, we observed a polarization toward the M2 profibrotic “resolver” macrophage phenotype in WT HOCl-mice, that was even more pronounced in *nrf2*^−/−^ HOCl-mice (**Figures 3K,L**, *p* = 0.044 and *p* = 0.049 for *nrf2*^−/−^ HOCl-mice versus WT HOCl-mice). Likewise, we observed an upregulation of the expression of M2 macrophages markers Fizz1, Arginase 1, and IL4R in the skin of *nrf2*^−/−^ HOCl-mice compared to WT HOCl-mice (**Figures 3N–P**, *p* = 0.0054, *p* < 0.0001, *p* = 0.0031). IL-13 can regulate the production of Abs by B cells and the polarization of macrophages toward the M2 phenotype (25). IL-13 also has profibrotic properties that can be crucial in the pathogenesis of SSc and is increased in the skin of WT-HOCl mice compared to untreated control animals. We observed a slight, but not significant, increase in IL-13 in *nrf2*^−/−^ HOCl-mice compared to WT-HOCl mice (*p* = 0.32, **Figure 3M**).

Altogether, these data comfort the predominant role of *nrf2* in the redox balance and the immune dysregulation in SSc HOCl-mice.

### The Nrf2 Agonist DMF Display Antioxidant Properties in Fibroblasts and Endothelial Cells

Antioxidant effects of DMF, a fumaric acid ester that display an agonist activity on *nrf2*, were evaluated *in vitro* in fibroblastic and endothelial cell lines and in murine and human primary diseased fibroblasts. A range of DMF doses were first tested *in vitro* on these different cell types (data not shown).

**Figure 4:**
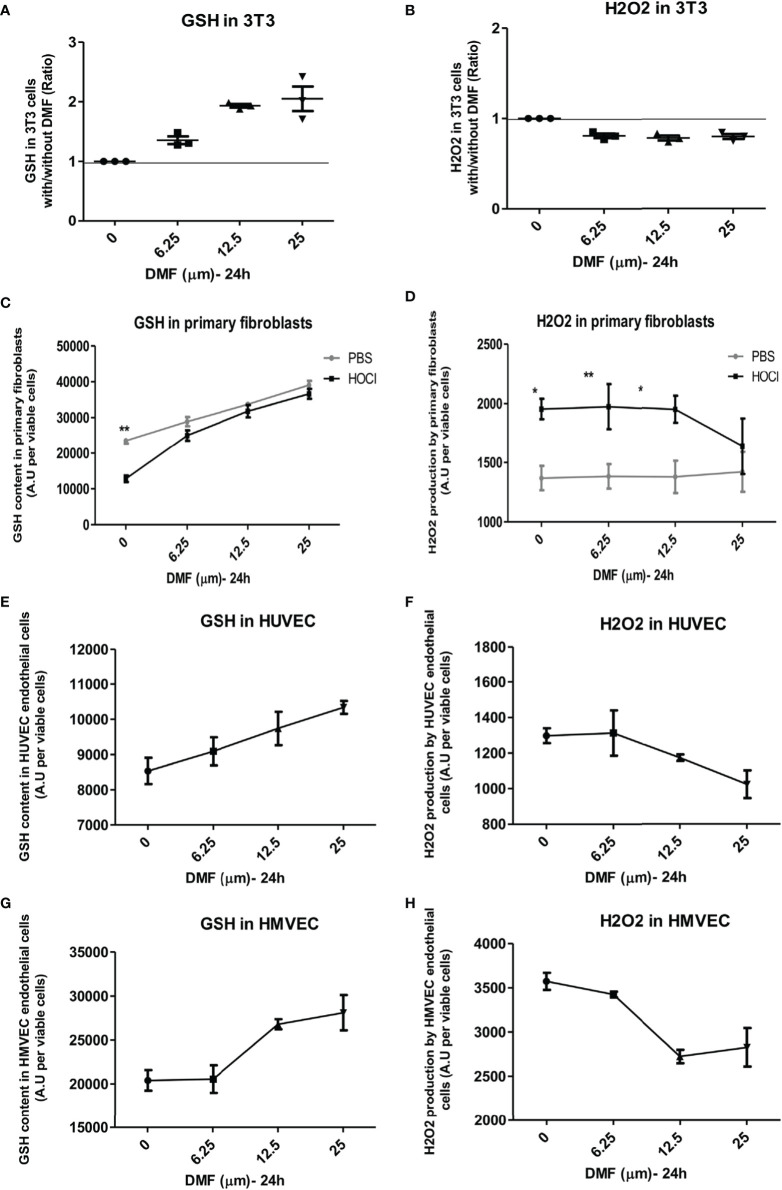
Dimethyl fumarate (DMF) has antioxidant properties *in vitro*. Cells were incubated *in vitro* with increasing concentrations of DMF for 24 h. Glutathione (GSH) content and H_2_O_2_ production were measured by spectrofluororometry in triplicates. **(A,B)** Effects of DMF on GSH content **(A)** and H_2_O_2_ production **(B)** in 3T3-fibroblasts, expressed as ratio with/without DMF. **(C,D)** Effects of DMF on GSH content **(C)** and H_2_O_2_ production **(D)** in phosphate buffered saline (PBS)- and HOCl-primary skin fibroblasts (from three mice per group). **(E,F)** Effects of DMF on GSH content **(E)** and H_2_O_2_ production **(F)** in human venal endothelial cells (HUVEC), expressed as ratio with/without DMF. **(G,H)** Effects of DMF on GSH content **(G)** and H_2_O_2_ production **(H)** in HMVEC endothelial cells, expressed as ratio with/without DMF. Values are mean ± SEM. *p ≤ 0.05; **p ≤ 0.01; by Mann–Whitney *U* test.

NIH-3T3-fibroblasts were treated *in vitro* with increasing amounts of DMF for 24 h. A dose-dependent elevation in GSH was observed: a dose of 6.25 µM DMF allowed an increase in GSH content by 40% and a concomitant decrease in H_2_O_2_ content by 20% (**Figures 4A,B**).

Primary skin fibroblasts from PBS- and HOCl-mice were also treated *in vitro* with increasing amounts of DMF. Fibroblasts from HOCl-treated mice showed a significant drop in GSH content compared to normal control fibroblasts (**Figure 4C**, *p* < 0.0001). A significant increase in GSH content was observed in primary fibroblasts from both PBS- and HOCl-mice upon DMF treatment, but this effect was amplified in fibroblasts from HOCl-mice as the content of GSH in those cells reached those of control PBS-fibroblasts and was no more significantly different at 6.25 and 12.5 µM DMF (**Figure 4C**) as was the levels of H_2_O_2_ between the two types of cells at 25 µM DMF (**Figure 4D**).

Endothelial cells represent a key target in SSc as many vascular abnormalities related to endothelial dysfunctions have been described (26). A dose-dependent increase in GSH content along with a decrease in H_2_O_2_ levels was observed in the endothelial cell lines HUVEC and HMVEC that was optimal at the dose of 25 µM DMF (**Figures 4E–H**). These results confirm the antioxidant effect of DMF in both fibroblasts and endothelial cells.

**Figure 5:**
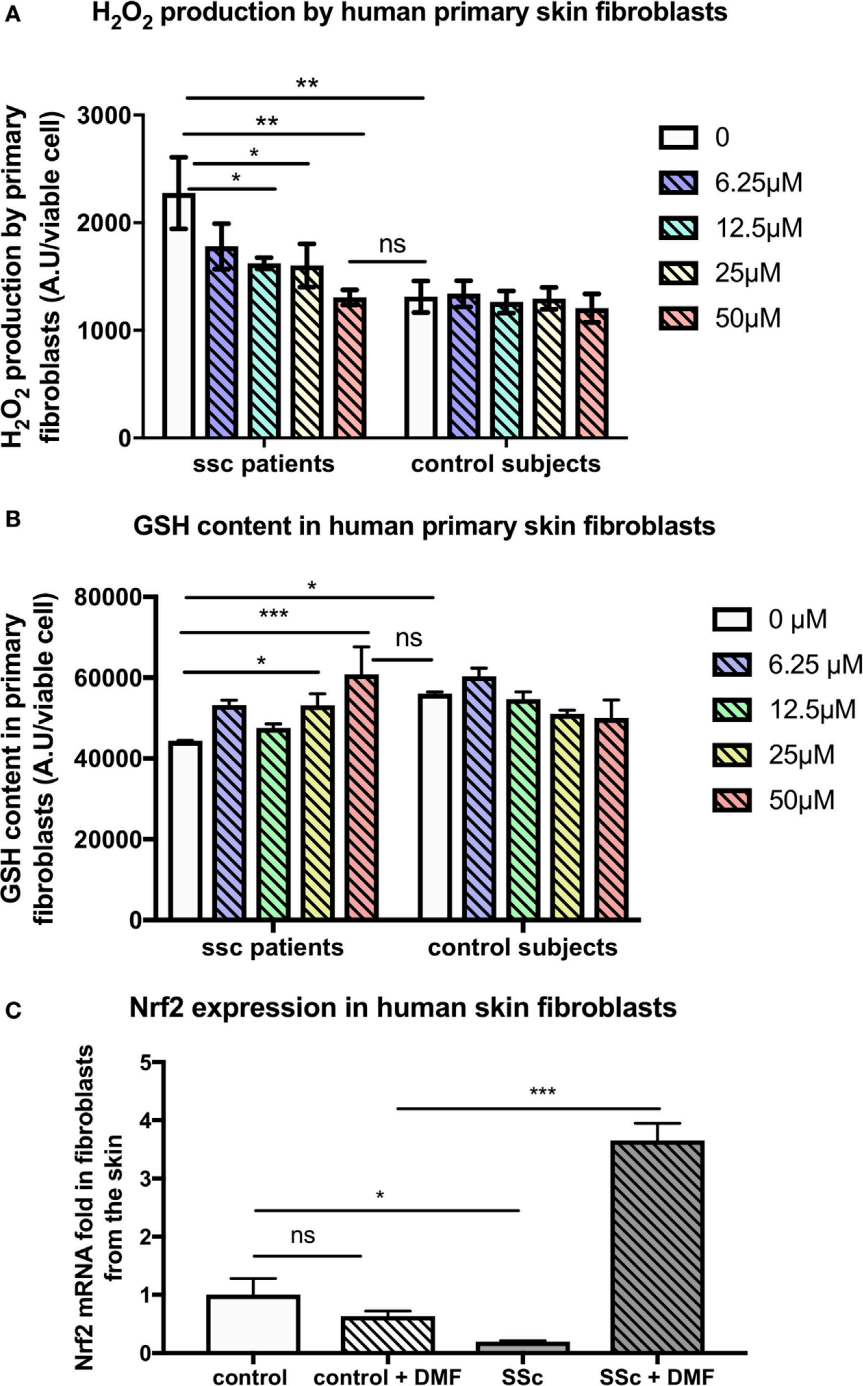
Skin fibroblasts from systemic sclerosis (SSc) patients display reduced H_2_O_2_ production and increased *nrf2* expression upon dimethyl fumarate (DMF) *in vitro* treatment. Human primary skin fibroblasts from SSc patients (*n* = 2, forearm skin biopsies) and control subjects (*n* = 2) were treated with increasing doses of DMF for 24 h. **(A)** H_2_O_2_ production of human skin fibroblasts treated with increasing doses of DMF (0–50 µM). **(B)** Glutathione (GSH) content in human skin fibroblasts treated with increasing doses of DMF (0–50 µM). **(C)** Expression of *nrf2* in skin fibroblasts upon *in vitro* treatment with 50 µM of DMF. Values are mean ± SEM. *p ≤ 0.05; **p ≤ 0.01; ***p ≤ 0.001, by Mann–Whitney *U* test

In addition, our findings were confirmed on human fibroblasts from SSc patients and control subjects. Indeed, DMF also exerted beneficial antioxidant effects on primary skin fibroblasts from SSc patients. DMF dose-dependently decreased the levels of H_2_O_2_ produced by skin fibroblasts from SSc patients and restored H_2_O_2_ production to the levels of production of control fibroblasts (**Figure 5A**). *In vitro* treatment with DMF also restored the GSH content in these cells (**Figure 5B**). Finally, *in vitro* treatment with 50 µM DMF of these human SSc skin fibroblasts significantly induced the expression of *nrf2* (**Figure 5C**).

### Treatment of HOCl-Mice With DMF Prevents the Development of SSc

**Figure 6:**
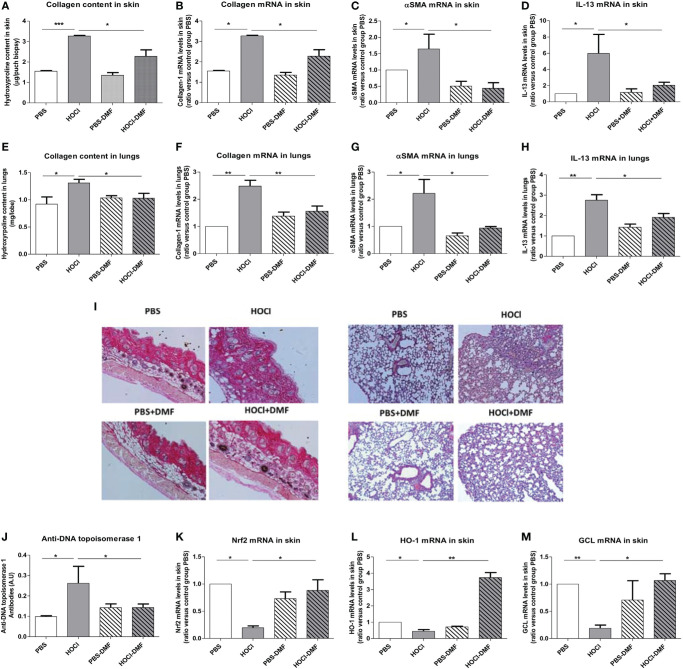
Effects of *in vivo* treatment with DMF on HOCl-induced SSc. BALB/c mice were intradermally injected with HOCl or phosphate buffered saline (PBS) and simultaneously treated with DMF or vehicle alone for 6 weeks. **(A–D)** *In vivo* treatment with DMF reduces skin fibrosis in mice with HOCl-induced SSc. **(A)** Collagen type I content in skin (Hydroxyproline, mg/punch biopsy). **(B)** Collagen type I mRNA levels in skin (ratio versus control group PBS). **(C)** Alpha-SMA mRNA levels in skin (ratio versus control group PBS). **(D)** IL-13 mRNA levels in skin (ratio versus control group PBS). **(E–H)** DMF reduces lung fibrosis in mice with HOCl-induced SSc. **(E)** Collagen type I content in lungs (Hydroxyproline, mg/lobe). **(F)** Collagen type I mRNA levels in lungs (ratio versus control group PBS). **(G)** Alpha-SMA mRNA levels in lungs (ratio versus control group PBS). **(H)** IL-13 mRNA levels in lungs (ratio versus control group PBS). **(I)** Skin and lung biopsies stained with Hematoxylin and eosin (H&E). Representative sections of 5 µm. Photographs were taken with a Nikon Eclipse 80i microscope. Original magnification ×50. **(J)** Anti-DNA topoisomerase 1 antibodies levels in the sera (A.U). **(K–M)** *In vivo* treatment with DMF upregulates the Nrf2 pathway in the skin. Levels of Nrf2 **(K)**, HO-1 **(L)**, and GCL **(M)** mRNAs in the skin (ratio versus control group PBS). Abbreviations: AU, arbitrary units; DMF, dimethyl fumarate; SSc, systemic sclerosis; HO-1, heme oxygenase-1. Values in **(A–M)** are mean ± SEM (*n* = 8 mice per group). *p ≤ 0.05; **p ≤ 0.01; ***p ≤ 0.001, by Mann–Whitney *U* test.

We explored the clinical *in vivo* effects of DMF in mice with SSc. Mice exposed to daily injections of HOCl developed increased skin and lung fibrosis with elevated collagen contents that were significantly reduced by *in vivo* treatment with DMF (*p* = 0.03 and 0.045, **Figures 6A,E**). Quantification of mRNA levels of collagen-1 (skin *p* = 0.045, lung *p* = 0.005, **Figures 6B,F**) and α-SMA (skin *p* = 0.04, lung *p* = 0.08, **Figures 6C,G**) confirmed the beneficial effect of DMF. Staining of skin and lung biopsies with Hematoxylin and Eosin also showed a reduction in fibrosis in both organs in diseased-mice treated with DMF compared to untreated diseased-mice (**Figure 6I**).

The development of fibrosis in the skin and lungs of HOCl-mice correlated with the elevation of the profibrotic cytokine IL-13 in these organs. Treatment with DMF decreased the levels of IL-13 in both skin and lungs from HOCl-mice (*p* = 0.02 in skin and *p* = 0.041 in lungs versus HOCl-mice; **Figures 6D,H**).

As mentioned above, mice exposed daily to HOCl develop an autoimmune reaction characterized by the presence of anti-DNA topoisomerase 1 autoantibodies. Treatment with DMF allowed a reduction in the development of these autoantibodies in mice with HOCl-induced SSc (*p* = 0.047 versus untreated HOCl-mice, **Figure 6J**).

Altogether, these data report a beneficial role of DMF in the treatment of SSc in mice, as the molecule ameliorates both fibrotic and inflammatory phenomenons *in vivo*.

We tested the clinical effects of DMF administered with the same dose and schedule in another mouse model of SSc induced by daily administration of bleomycin for 6 weeks. Consistent with the results in the HOCl model of SSc, histopathological studies of skin sections stained with picro-sirius red showed that the excess of collagen accumulation in the skin of bleomycin-injected mice returns to normal upon DMF treatment. This observation was confirmed by the significant reduction in the expression of the fibrotic markers α-SMA (*p* = 0.001) and type-1 collagen (*p* = 0.05) in the skin of bleomycin-injected mice treated with DMF compared to untreated animals (Figure S1 in Supplementary Material).

### Treatment With DMF Induces an Nrf2 Signature in Skin Fibroblasts From Mice With HOCl-Induced SSc

We investigated whether the amelioration of the clinical symptoms of SSc was related to a reduction in oxidative stress markers in mice. *In vivo* DMF treatment of HOCl-mice allowed an elevation of the transcription levels of *nrf2* in the skin along with an increase in its target genes GCL and HO-1 (*p* = 0.013, *p* = 0.019, and *p* = 0.002, respectively, versus untreated HOCl-mice, **Figures 6K–M**).

These data confirm that the clinical amelioration of the symptoms of SSc observed in mice following DMF treatment are related to the enhanced transcription of Nrf2 and its downstream target genes HO-1 and GCL triggering a cytoprotective and antioxidant response.

## Discussion

It is now well established that *nrf2* plays important roles in the cellular adaptive defense responses to oxidative stress, leading to an efficient expression of detoxifying enzymes and antioxidant molecules. In this study, we report a defect in the *nrf2* pathway in patients and mice with SSc, an autoimmune disease with fibrosis and vascular dysfunctions. We also bring new evidence for a direct role of this defect in the induction of the disease using murine models of SSc, *nrf2* knockout mice, and *in vivo* treatment with DMF, an agonist of *nrf2*.

Our cohort of SSc patients displayed a downregulation of the *nrf2* pathway (i.e., *nrf2* and *nrf2*-target genes GCL, HO-1, and TRX) in fibroblasts from fibrotic skin. We observed the same results in fibrotic skin and lungs from mice with HOCl-induced SSc. These results provide new insights on the role of intrinsic ROS dysregulation in SSc fibroblasts and strengthen the link between *nrf2* and fibrosis, in accordance with the recent findings from Wei et al. (27). Indeed, the drop in antioxidant levels and the overproduction of ROS have previously been described in scleroderma and assigned with a direct role in the pathogenesis of SSc as ROS can trigger the proliferation of fibroblasts and collagen synthesis (28, 29). However, so far, the intrinsic mechanism responsible for the redox imbalance in SSc fibroblasts remains unclear. The drop in NRF2 levels in the skin of SSc patients may be a major factor at the origin of this imbalance between oxidant and antioxidant molecules in scleroderma. HOCl-mice, as well as SSc patients, display an important depletion in GSH in skin fibroblasts that is responsible for the uncontrolled ROS production and proliferation of these cells leading to fibrosis (7). Our present data bring new insights to explain this phenomenon as the downregulation of NRF2 in SSc fibroblasts can directly lead to the lack of GSH through the drop in GCL expression, its rate-limiting enzyme (30, 31). NRF2 levels have indeed been shown to directly regulate the levels of GSH in many cellular types both in normal and pathologic conditions such as cancer (32, 33). Thus, in SSc fibroblasts, the downregulation of NRF2 will lead to a drop in GSH concentration and to an uncontrolled ROS production. Furthermore, elevated ROS levels will induce an increased phosphorylation of the MAPK pathway proteins (34) leading to fibroblasts proliferation, along with an activation of the Smad pathway, which subsequently leads to collagen production (35).

We next showed that the SSc features were exacerbated in *nrf2* KO-mice when inducing SSc by daily exposure to HOCl as demonstrated by the major defects in antioxidative defenses, increased fibrosis of skin and lungs and immune activation with inflammation and autoimmunity. Recently, Wei et al. also reported that *nrf2* KO-mice displayed an exacerbated phenotype of bleomycin-induced SSc (27). *Nrf2*-KO-mice are more prompt to develop fibrotic and inflammatory responses, and the daily exposure to HOCl emphasized this phenomenon (20, 36). Indeed, in endometriosis, a gynecological disease with dysregulated proliferation of endometrial stromal cells closely resembling that of the dysregulated proliferation of fibroblasts in SSc, wild-type mice bearing endometrial implants from *nrf2*-KO-horn display an aggravated phenotype, with elevated volume of the implants and higher amounts of collagen and inflammatory markers compared to those with implants from wild-type animals (37). SSc is a systemic autoimmune disease with chronic inflammation and activation of adaptive immunity by nuclear auto-antigens such as DNA topoisomerase 1 and production of autoantibodies. Anti-DNA topoisomerase 1 Abs were detected in the sera from both WT-HOCl- and *nrf2*^−/−^ HOCl-mice but at a significantly higher titer in the sera from *nrf2*^−/−^ HOCl-mice compared to WT-HOCl-mice. Few data are available regarding *nrf2*-KO-mice in the context of autoimmunity. It has, however, been shown that autoimmunity in SSc may be dependent on the formation of ROS-induced oxidized neoepitopes that may induce the breach of tolerance against DNA topoisomerase I and autoimmunity (4). Thus, further increasing ROS by *nrf2* invalidation could be responsible for the overproduction of oxidized neoepitopes and autoantibodies.

In SSc pathogenesis, macrophages polarization plays a key role linking immune activation with fibrosis (38). The preponderance of M2 “resolver” macrophages over M1 both in the spleen and in the skin of *nrf2*-KO mice could also contribute to the enhanced phenotype of the disease in these animals. The exacerbated immune response observed in HOCl-*nrf2* KO-mice has also been reported in the model of experimental autoimmune encephalomyelitis where *nrf2* KO-mice show a more severe form of the disease compared to wild-type mice (39).

To confirm the pathogenic role of the *nrf2* defect in SSc, we tested the impact of its pharmacological activation with DMF, a potent and FDA-approved Nrf2 pharmacological activator.

In fibroblastic and endothelial cell lines, the two cell types particularly involved in ROS-mediated SSc pathogenesis, DMF shows potent antioxidative properties as demonstrated by the dose-dependent increase in the reduced GSH content of those cells and their reduced H_2_O_2_ production. We observed this effect on cell lines (3T3, HUVEC, and HMVEC), but also on primary murine and human cells from skin biopsies (**Figures 4** and **5**). At a dose of 50 µM, DMF strongly induced the expression of the *nrf2* gene in fibroblasts from SSc patients. Such an antioxidative effect was already observed by Hoffmann et al. who demonstrated that DMF was able to restore the GSH pool even in the context of total GSH depletion (40) as observed in fibroblasts from humans or mice with SSc. Increasing GSH was associated with a decreased production of ROS and a decreased proliferation of these cells.

The *in vivo* use of DMF in HOCl-induced SSc was associated with the induction of Nrf2 and the expression of its downstream antioxidant defense genes expression (HO-1 and GCL) along with a reduced skin and lung fibrosis and immune activation in DMF-treated HOCl-SSc mice compared to untreated animals. DMF restored to normal the content of collagen in the lung whereas its effect on the skin was much milder. Skin fibroblasts may need a higher dose of DMF than lung-fibroblasts to decrease more potently their proliferation and production of matrix. The Nrf2-activating properties of DMF have been widely studied but few data are available regarding its direct anti-fibrotic properties (41, 42). In human fibroblasts, DMF can promote the degradation of β-catenin, a transcriptional factor activating the Wnt pathway, implicated in pulmonary fibrosis (43). Wnt is a key mediator of fibrosis in HOCl-induced SSc, and besides its antioxidant properties, DMF could block directly the development of fibrosis through its regulating effect on Wnt (44). Toyama et al. have also recently studied the effect of DMF in SSc fibroblasts (43). Consistent with our results, they demonstrate that *in vitro* treatment with DMF can block the TGF-β-induced profibrotic response in fibroblasts *via* the inhibition of PI3K/Akt pathway and the transcriptional regulators TAZ and YAP (43).

In our work, the levels of anti-DNA topoisomerase 1 were significantly decreased in the sera of DMF-treated SSc mice compared to untreated SSc mice. This result is consistent with the *in vivo* elevation of antioxidant defenses induced by DMF treatment that blocks chronic ROS production and consequently the release of oxidized antigens that play a key role in the breach of immune tolerance leading to the development of the autoimmune reaction in SSc. DMF also displays direct anti-inflammatory properties including inhibition of NFκB and STAT3 pathways that are known to contribute to the inflammation in the HOCl-induced model of SSc (44). A recent paper has provided mechanistic insights into the immune-modulatory effects of DMF by showing that DMF can inhibit the aerobic glycolysis in activated immune cells (45).

In summary, we confirm the profound defect of the Nrf2 antioxidant pathway in skin fibroblasts of SSc patients and mice, together with the aggravation of the disease in *nrf2*-KO SSc mice. We propose a critical regulatory role for Nrf2 in the homeostasis of oxidative stress in SSc. Recently published results regarding the role of Nrf2 in other models of SSc and the use of DMF in pulmonary arterial hypertension models strongly confirm our observations and, along with ours, brings major data to strongly support the use of DMF to ameliorate the clinical symptoms of SSc, as the molecule has already been approved by the FDA in multiple sclerosis and psoriasis (27, 42, 43).

## Ethics Statement

Animal (mice): this study was carried out in accordance with the recommendations of the Regional Ethic Committee on Animal Experimentation under the number CEEA34.CN.023.11. The protocol was approved by the Regional Ethic Committee on Animal Experimentation. Human samples: the samples were collected by the Rheumatology Department of Cochin Hospital. This study was carried out in accordance with the recommendations of the Cochin Hospital Ethic Committee. The protocol was approved by the Cochin Hospital Ethic Committee. All subjects gave written informed consent in accordance with the Declaration of Helsinki.

## Author Contributions

Conceived and designed the experiments: NK, FB, SM, and YA. Performed the experiments: NK, SM, MJ, CN, CC, CCh, MA-D, and SC. Analyzed the data: NK, SM, FB, SK-R, and YA. Contributed reagents/materials/analysis tools: NK, FB, NS, OC, AC, SC, SK-R, and YA. Wrote the paper: NK, FB, and SM.

## Conflict of Interest Statement

The authors declare that the research was conducted in the absence of any commercial or financial relationships that could be construed as a potential conflict of interest.

## Supplementary Material

Figure S1 *In vivo* treatment with dimethyl fumarate (DMF) reduces bleomycin-induced fibrosis. BALB/c mice were injected subcutaneously with bleomycin or phosphate buffered saline (PBS) and simultaneously treated with DMF (“Bleo DMF”) or vehicle alone (“Bleo”) for 6 weeks. **(A,B)** Alpha-SMA and collagen type 1 mRNA levels in the skin of PBS- and bleomycin-mice. **(C)** Skin biopsies stained with picro-sirius red. Representative sections of 5 µm. Photographs were taken with a Nikon Eclipse 80i microscope. Original magnification ×50. Values in **(A,B)** are mean ± SEM (*n* = 7 mice per group). **p* ≤ 0.05; ***p* ≤ 0.01, by Mann–Whitney *U* test.

Table S1 Primers used for quantitative RT-PCR in this study.

## Abbreviations

ROS, reactive oxygen species; SSc, systemic sclerosis; Nrf2, nuclear factor erythroid 2 (NF-E2)-related factor 2; GSH, glutathione; GCL, glutamate cystein ligase; HO-1, heme oxygenase-1.
