# Corrigendum: The *Nrf2*-Antioxidant Response Element Signaling Pathway Controls Fibrosis and Autoimmunity in Scleroderma

**DOI:** 10.3389/fimmu.2021.737303

**Published:** 2021-07-27

**Authors:** Niloufar Kavian, Souad Mehlal, Mohamed Jeljeli, Nathaniel Edward Bennett Saidu, Carole Nicco, Olivier Cerles, Sandrine Chouzenoux, Anne Cauvet, Claire Camus, Mehdi Ait-Djoudi, Christiane Chéreau, Saadia Kerdine-Römer, Yannick Allanore, Frederic Batteux

**Affiliations:** ^1^Laboratoire d’Immunologie, Hôpital Cochin, Paris, France; ^2^INSERM U1016, Institut Cochin, Paris, France; ^3^UMR996 - Inflammation, Chemokines and Immunopathology, INSERM, Univ Paris-Sud, Université Paris-Saclay, Châtenay-Malabry, France; ^4^Service de Rhumatologie, Hôpital Cochin, Paris, France

**Keywords:** systemic sclerosis, oxidative stress, fibrosis, inflammation, Nrf2

In the original article, there was a mistake in the legend for **Figure 3G** and **6I** as published. Skin and lung biopsies were stained with Haematoxylin and Eosin, and not “with picro-sirius red and Haematoxylin and Eosin” as stated in the original legend. The correct legends appear below. The authors apologize for this error and state that this does not change the scientific conclusions of the article in any way. The original article has been updated.

**Figure 3G:**
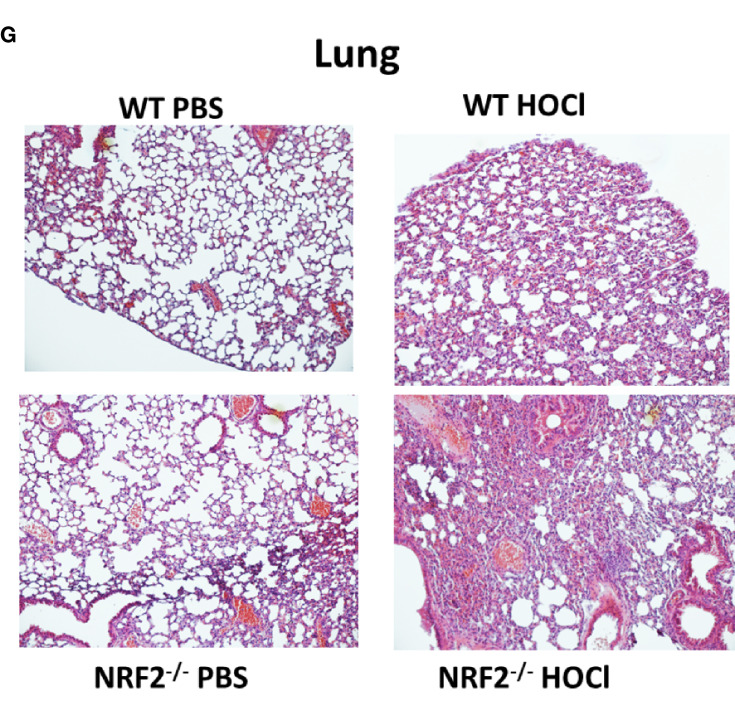
**(F, G)** Skin and Lung biopsies stained with Hematoxylin and eosin (H&E).

**Figure 6I:**
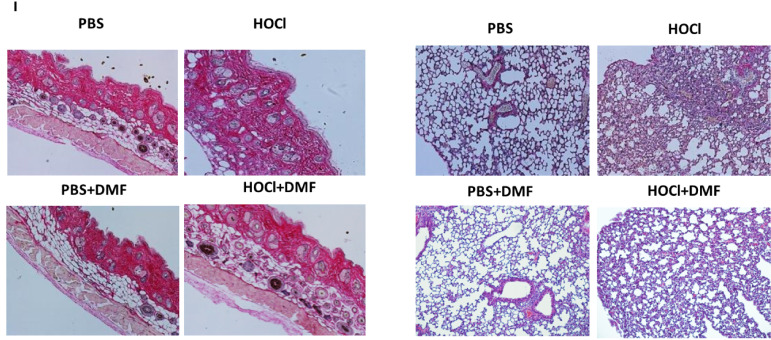
**(I)** Skin and lung biopsies stained with Hematoxylin and eosin (H&E).

In the original article, there was a mistake in the photos used in **Figures 3G** and **6I** as published. The authors noticed that some of the lung histology photos used in **Figures 3** and **6** as illustrations only were not correct due to an inadvertent mishandling of the names list and photo files. The corrected **Figures 3** and **6** appear below. The authors apologize for this error and state that this does not change the scientific conclusions of the article in any way, as these photos were used as illustrations only, and not as a source of quantifiable data. The original article has been updated.

In the original article, there was an error in the **Methods** section: “A 5-μm-thick tissue section was prepared from the mid-portion of paraffin-embedded tissue and stained with H&E or picro-sirius red.”

A correction has been made in the text of the **Methods** section**, **Assessment of Skin Thickness and Collagen Accumulation in Skin and Lungs**, paragraph 1:

“A 5-μm-thick tissue section was prepared from the mid-portion of paraffin-embedded tissue and stained with H&E.”

In the original article, there was an error in the **Results** section: “Staining of skin and lung biopsies with picro-sirius red also showed a reduction in fibrosis in both organs in diseased-mice treated with DMF compared to untreated diseased-mice (**Figure 6I**).”

A correction has been made in the text of the **Results** section, **Treatment of HOCl-mice with DMF prevents the development of SSc**, paragraph 1:

“Staining of skin and lung biopsies with Hematoxylin and Eosin also showed a reduction in fibrosis in both organs in diseased-mice treated with DMF compared to untreated diseased-mice (**Figure 6I**).”

The authors apologize for this error and state that this does not change the scientific conclusions of the article in any way. The original article has been updated.

## Publisher’s Note

All claims expressed in this article are solely those of the authors and do not necessarily represent those of their affiliated organizations, or those of the publisher, the editors and the reviewers. Any product that may be evaluated in this article, or claim that may be made by its manufacturer, is not guaranteed or endorsed by the publisher.

