# Supplementary figures and images for: The Nrf2-Antioxidant Response Element Signaling Pathway Controls Fibrosis and Autoimmunity in Scleroderma
Source: Front Immunol. 2018 Aug 16;9:1896. doi: 10.3389/fimmu.2018.01896 (PMC6109691; doi:10.3389/fimmu.2018.01896)

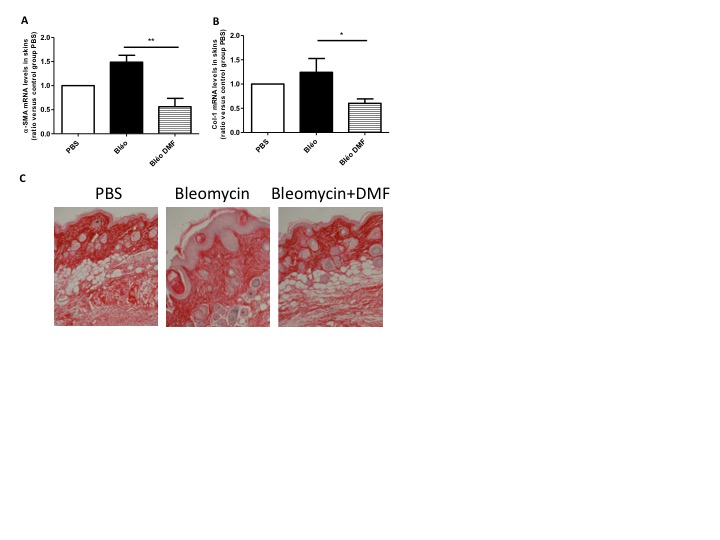

Supplement: Figure S1 — In vivo treatment with dimethyl fumarate (DMF) reduces bleomycin-induced fibrosis. BALB/c mice were injected subcutaneously with bleomycin or phosphate buffered saline (PBS) and simultaneously treated with DMF (“Bleo DMF”) or vehicle alone (“Bleo”) for 6 weeks. (A,B) Alpha-SMA and collagen type 1 mRNA levels in the skin of PBS- and bleomycin-mice. (C) Skin biopsies stained with picro-sirius red. Representative sections of 5 µm. Photographs were taken with a Nikon Eclipse 80i microscope. Original magnification ×50. Values in (A,B) are mean ± SEM (n = 7 mice per group). *p ≤ 0.05; **p ≤ 0.01, by Mann–Whitney U test. [file Image_1.jpeg]
