# Supplementary material for: The Nrf2-Antioxidant Response Element Signaling Pathway Controls Fibrosis and Autoimmunity in Scleroderma
Source: Front Immunol. 2018 Aug 16;9:1896. doi: 10.3389/fimmu.2018.01896 (PMC6109691; doi:10.3389/fimmu.2018.01896)
Supplement: Table S1 — Primers used for quantitative RT-PCR in this study. [file Table_1.pdf]

Murine primers		
**Gene**	**Forward (5'-3')**	**Reverse (5'-3')**
**aSma**	AAGGCCAACCGGGAGAAAAT	AGCCAAGTCCAGACGCATGA
**Col1**	TGTTCAGCTTTGTGGACCTC	TCAAGCATACCTCGGGTTTC
**IL13**	GCTTGCCTTGGTGGTCTCGCC	GGGCTACACAGAACCCGCCA
**Nrf2**	CTCGCTGGAAAAAGAAGTG	CCGTCCAGGAGTTCAGAGG
**HO1**	CACGCATATACCCGCTACCT	CCAGAGTGTTCATTCGAGA
**GCL**	TGGAGCAGCTGTATCAGTG	AGAGCAGTTCTTTCGGGTA
**Arginase1**	TGGCTTGCGAGACGTAG	GCTCAGGTGAATCGGCC
**Fizz-1**	TATGAACAGATGGGCCT	CCACTCTGGATCTCCCA
**IL4-R**	TCTGCATCCCGTTGTTTT	GCACCTGTGCATCCTGA
**HRPT2**	CCCCCCCTTGAGCACACAG	TGCCGAGGATTTGGAAAAAG

Human primers		
**Gene**	**Forward (5'-3')**	**Reverse (5'-3')**
**Nrf2**	CATCCAGTCAGAAACCAGTGG	GCAGTCATCAAAGTACAAAGCAT
**HO-1**	CTTCAAGCTGGTGATGGCC	GAAGTAGACAGGGGCGAAGA
**GCL**	GGCACAAGGACGTTCTCAAG	CTGTCCTGGTGTCCCTTCAA
**GAPDH**	GCCACATCGCTCAGACAC	GCCCAATACGACCAAATCC
